# FAK-Dependent Cell Motility and Cell Elongation

**DOI:** 10.3390/cells9010192

**Published:** 2020-01-12

**Authors:** Kazuo Katoh

**Affiliations:** Laboratory of Human Anatomy and Cell Biology, Faculty of Health Sciences, Tsukuba University of Technology Tsukuba-city, Ibaraki 305-8520, Japan; katoichi@k.tsukuba-tech.ac.jp; Tel.: +81-29-858-9557

**Keywords:** FAK, focal adhesion, c-Src, cell motility, cell elongation

## Abstract

Fibroblastic cells show specific substrate selectivity for typical cell–substrate adhesion. However, focal adhesion kinase (FAK) contributes to controlling the regulation of orientation and polarity. When fibroblasts attach to micropatterns, tyrosine-phosphorylated proteins and FAK are both detected along the inner border between the adhesive micropatterns and the nonadhesive glass surface. FAK likely plays important roles in regulation of cell adhesion to the substrate, as FAK is a tyrosine-phosphorylated protein that acts as a signal transduction molecule at sites of cell–substrate attachment, called focal adhesions. FAK has been suggested to play a role in the attachment of cells at adhesive micropatterns by affecting cell polarity. Therefore, the localization of FAK might play a key role in recognition of the border of the cell with the adhesive micropattern, thus regulating cell polarity and the cell axis. This review discusses the regulation and molecular mechanism of cell proliferation and cell elongation by FAK and its associated signal transduction proteins.

## 1. Introduction

When cultured on a glass surface, the plasma membrane of fibroblastic cells begins to move from the distal end to the leading edge [1]. The morphology of the cell membrane is deformed via the depolymerization of the actin cytoskeleton, such that the focal adhesions between the extracellular matrix (ECM) and intracellular proteins move forward to the leading edge [2,3]. The plasma membrane and its associated focal adhesions at the rear of the cell are destroyed by the activation of specific kinases, being referred to as focal adhesion kinase (FAK) [4,5,6]. The cells form multiple proturusions when the cell is moving. Polymerisation and bundling of linear actin filaments within fan like lamellipodia forms actin filaments-based protrusions, named filopodia, and Src and FAK seems to control pathways that lead to their formation. Filopodia can align along with focal adhesions, but it is not clear whether the filopodial actin structure is force generating, or whether the role is more closely linked to cell elongation. The localization of receptors and adhesion molecules, such as integrins, is known to be highly polarized when cells are moving directionally in culture. Integrins have been implicated in cellular migration in many contexts [5]. The polymerization of actin filaments organize protrusions that are provided by membrane tension to specify cell shape. Cell adhesion and locomotion are membrane based processes. The cell membranes are composed of the plasma membrane, which is mechanically stabilized by a thick macromolecular network that is composed of the actin filaments. Actin filaments are locally attached to the intracellular domains of the integrins. To push the cell front forward, the protrusion force has to be balanced by shear deformation of the substrate in the opposite direction [7]. The integrins are focal adhesion proteins, through which the ECM interacts with the internal environment of the cells. Integrins are dimeric transmembrane proteins that consist of α and β subunits localized at focal adhesions, which act as signaling molecules between the ECM and the plasma membrane [3,8,9,10,11,12,13]. Controlling cellular adhesion, the turnover of integrins by endocytosis or exocytosis is necessary for cell movement [14]. This seems to be controlled by FAK and associated substrates [15], including the Src family of tyrosine kinases (SFK) [3]. SFK is a family of oncogenes, which were discovered in association with cancer. The tumors in chickens were shown to be caused by the Rous sarcoma virus oncogene, v-Src, which is similar to the typical cellular protein, c-Src, but is lacking the C-terminus. Unlike c-Src, v-Src is constitutively active, as it lacks the C-terminal inhibitory phosphorylation site (Y527) [16]. The c-Src protein is a signaling molecule that is involved in controlling cell growth, proliferation, and/or motility. FAK was shown to be important for cell migration, as Src-deficient cells showed reduced motility [17]. Cells that were deficient in c-Src might be linked in signaling by extracellular matrix-coupled receptors, such as integrins [18]. Src is present on the intracellular side of the plasma membrane and it regulates focal adhesion-associated proteins, including FAK and paxillin, as well as proteins that are known to mediate cytoskeletal remodeling.

The c-Src protein is a signaling protein that is involved in the regulation of the growth, proliferation, and/or motility of cells. This protein is only present in the intracellular side of the plasma membrane, where it is involved in the “ON/OFF switch” from the outside of the cell. The organization of the cytoskeleton that is involved in controlling membrane protrusion during cell movement appears to be under the control of c-Src and FAK, as the cell motility is inhibited in c-Src knockout mice [19]. In addition, c-Src and FAK both mediate signal transduction from the external environment to the inside of the cell, and regulate cell proliferation [20]. Various functions are necessary for the signal transduction mechanisms that are involved in cell adhesion to a rigid substrate.

FAK and c-Src are intracellular (nonreceptor) tyrosine kinases that physically and functionally interact to promote a variety of cellular responses [20,21,22]. The ability to promote tumor cell proliferation suggests a central role of this complex in cancer. It has also been established that the FAK–Src complex plays an important role in tumor angiogenesis. FAK and Src are associated with solid tumor metastasis through their ability to promote epithelial–mesenchymal transition. Indeed, a strong correlation has been reported between increased FAK/Src expression/phosphorylation and the invasive phenotype of human tumors. New anticancer drugs targeting FAK or Src are currently under development for a wide variety of tumors. The normal cellular function of the FAK–Src complex has been reported to include acting as an effector of integrin and/or tyrosine kinase receptor signaling [23]. FAK activation involves integrin receptor accumulation and binding to the ECM proteins, which results in the dimerization of FAK [24]. This FAK dimerization induces autophosphorylation of FAK at pY397, resulting in the Src-mediated phosphorylation of the FAK kinase domain (pY576 or pY577), thus leading to the formation of the active FAK–Src complex [23,25].

There have been many studies of the relations between c-Src, Fyn, and c-Yes, all of which are members of the SFK, since the discovery of v-Src and its associated FAK. As FAK is localized in focal adhesions, together with paxillin, Crk-related substrate (Cas), talin, vinculin, etc., the focal adhesions and related cell scaffold proteins were identified as proteins that connect the ECM to the internal cytoskeleton [26]. Small focal adhesion-like structures, including FAK, are localized at the leading edge of the cell membrane in cultured fibroblasts. The integrins, which are cell adhesion receptors, combine with components of the ECM (e.g., fibronectin and/or collagen), resulting in signal transmission to SFK (Fyn and c-Src), which lead to their activation. FAK is activated and it undergoes autophosphorylation, and then accelerates phosphorylation of paxillin and Cas. The proteins that interact with the SH2 domain of paxillin and Cas accumulate and form a large protein complex at well-developed focal adhesions [27].

The focal adhesion plays essential roles in many cellular events, including cell migration, wound healing, and angiogenesis.. Focal adhesions are discrete adhesive plaques that contain numerous structural (e.g., vinculin, talin, and α-actinin) and signaling molecules (e.g., FAK, Src, paxillin, p130Crk-related substrate, RhoA, and integrins) [26]. Some signal transduction proteins, such as FAK, c-Src, Rho A, and integrin, are localized along with these proteins in close association with focal adhesions. These observations strongly suggest that the focal adhesions play roles not only in the connection between the plasma membrane of the cell and substrate, but also in transferring specific signals from the outside to the inside of the cell. The focal adhesions recognize the boundary between the plasma membrane and suitable ECM proteins, together with the stress fibers, and such focal adhesions also determine cell orientation and polarity [28]. Fibroblastic cells show specific substrate selectivity for typical cell–substrate adhesion, and FAK seems to control the mechanisms of initial contact with the particular substrate that regulate their direction and polarity [29].

Cell migration contributes to a number of pathological processes, including vascular disease, osteoporosis, chronic inflammatory disease, and cancer [30,31]. Actin-containing microfilaments form stress fibers that are polarized with fast growing barbed plus ends and slow growing pointed minus ends, and this polarity results in polarized membrane protrusion. Actin filaments form a branching network at the fibroblast cell front. The Arp2/3 complex mediates the polymerization of actin in areas of projection at the leading edge of the cell called lamellipodia [32,33]. Cell migration is also controlled by the formation of focal adhesions in the front region of the cell. In the rear portion of the cell, detachment of focal adhesions occurs simultaneously, exhibiting reduced trailing edge retraction, which results in cell elongation. FAK, Rho-GEF, and Rho kinase II (ROCKII) are crucial for the regulation of adhesion movement and trailing edge retraction [31]. Focal adhesion at the leading edge of the cell is important for determination of the cell axis and cell elongation. When cells begin to migrate, small focal adhesion-like structures become organized in protrusions at the front of the cell, and well-developed stress fibers form along the longitudinal axis of migrating cells [29]. Focal adhesions at the front of the cell form adhesion foci, which regulate the direction of migration with the migration-promoting receptor proteins, i.e., the integrins, and promote inside-out signaling at the focal adhesions [34]. The knock-down of FAK expression induces cell elongation [34,35] and it results in defects in focal adhesion disassembly [36]. The number of focal adhesions was shown to be increased in FAK-deficient cells, which suggests that FAK might be involved in the turnover of focal adhesion contacts during cell migration [37]. FAK seems to play roles in the disruption of focal adhesions during cell migration, as it disrupts the foot points of adhesion foci and seems to determine cell elongation and the direction of migration.

## 2. Localization of Integrins and FAK at Focal Adhesions and Its Interaction within in the Cell

FAK is localized at focal adhesions and it aggregates to the cytoplasmic tails of integrins. FAK binds directly to paxillin [38,39], and it has also been shown to be localized to the endosomes. Endocytic trafficking of integrins represents an important complementary mechanism for regulating integrin–ECM adhesion turnover and receptor recycling [13]. Phosphorylated FAK is colocalized with β1 integrin, talin, and Rab GTPases on the early endosomes [40]. In addition, FAK has also been shown to be localized at sites of cell–cell adhesion, called adherens junctions formed by cadherins, catenins, and α-actinin, in epithelial cells and endothelial cells [41]. The translocation of FAK to the nucleus, where it promotes cell proliferation, has also been reported [42]. The findings outlined above strongly suggest that FAK has multiple physiological functions in cells. FAK can act as a platform for protein complex formation or as a bridge between proteins, and it is also known to function as a tyrosine kinase that is involved in the phosphorylation of different targets in several types of cells [43].

The integrins are a family of cell-surface proteins that interact with the ECM and transduce intracellular signals in response to information from the ECM to induce changes in cell shape/motility and cell cycle progression. Integrin receptors are composed of α and β subunits and they constitute structural and functional bridges between the ECM and intracellular backbone linker proteins [44]. Signaling that is mediated by integrin–ECM interaction is also integrated with cellular responses to signaling by growth factors. The cellular response regulates cell proliferation, cytoskeleton reconstitution, and other reactions that are required for cell survival.

Integrin receptors do not have a kinase domain but can adhere to the ECM and interact with multiple intracellular signaling pathways. When bound to the ECM, integrins bind to fibronectin, laminin, collagen, tenascin, vitronectin, thrombospondin, etc. Integrin mediates bidirectional “inside-out” signaling, which activates the ECM binding function of integrins, and “outside-in” signaling mediates cellular responses that are induced by the binding of ECM to integrins leading to cell spreading, retraction, migration, and proliferation. Along with the interaction between the integrin cluster and the ECM, cytoskeletal components, and intracellular signaling molecules accumulate to form adhesion foci. The cytoplasmic tail of integrins acts as a binding site for α-actinin and talin, which subsequently take up vinculin, a protein that binds F-actin to the cell membrane [45]. In addition to actin polymerization/depolymerization, ligand binding to the integrin receptor causes FAK talin-mediated oligomer formation. The auto-phosphorylation at Tyr397 of FAK enables the binding and activation of Src and Fyn, which in turn phosphorylate the FAK-associated proteins, paxillin, tensin, and p130Cas. Phosphorylated FAK phosphorylates and induces the activation of PI3K, PLCγ, and GRB7 [3]. The activation of PI3K causes the activation of integrin, which leads to activation of the cell survival mechanism in the Akt signaling pathway [46,47].

The phosphorylation of Tyr925 of FAK by c-Src results in the formation of a complex of GRB 2 and SOS, and activation of Ras. Ras, in turn, plays a role in activating various kinases, including MEKK, PAK, MEK, JNK, and SAPK, which control gene expression by phosphorylating many transcription factors, including c-Myc, Elk1, Jun, and serum response factor (SRF). Activated Src also phosphorylates p180Cas and induces the formation of a protein complex between Crk and DOCK 180, which is also localized to focal adhesions [44,48]. This protein complex enhances the membrane affinity of Rac and further activates the above-mentioned kinase pathway.

## 3. FAK as a Mediator of Mechanotransduction

FAK is one of the first molecules recruited to focal adhesions in response to external mechanical stimuli. The mechanosignal transduction that senses and organizes the information depends on the type of mechanical stimulation, and it is transmitted by the intracellular signaling proteins. Ion channels that sense mechanosignaling detect changes in plasma membrane tension [49,50,51]. The mechanical properties of the ECM are recognized by focal adhesions, mainly by the integrins, which are a class of transmembrane receptor proteins [52].

The actomyosin system that is localized to the inner region of focal adhesions not only generates contractile force [53], but also senses mechanical force [54,55,56]. The actomyosin system seems to act as a mechano-signal transduction sensor in focal adhesions [57], which strongly supports our previous notion that stress fibers play a role in mechano-signal transduction in endothelial cells [58,59].

**Figure 1:**
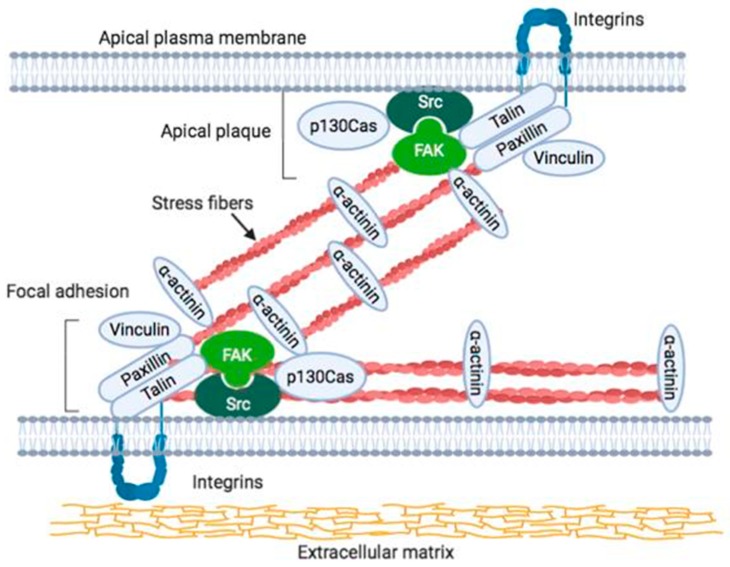
Schematic illustration of apical plaque and focal adhesion. Extracellular matrix, integrin, and cytoskeletal proteins, such as talin, paxillin, and vinculin, are localized in both the apical plaque and the focal adhesions. FAK–Src complex is also localized in both the apical plaque and the focal adhesions at the basal portion of the cell, which indicates that the complex acts as signal transduction machinery. Localization of FAK in apical plaque is very few or not detected [60].

Focal adhesion-like structures, called “apical plaques”, are localized on the apical side of endothelial cells directly facing the blood flow [58,60]. These structures are composed of the same proteins as focal adhesions, but they are localized on the free surface of the cell (i.e., on the luminal surface of blood vessels and renal artery) in situ cells and fibroblastic cells in vitro [58,59,61]. Furthermore, stress fibers that pass directly through the upper surface of the cell are connected to this apical plaque (Figure 1) [59]. On the other hand, focal adhesions that are present on the basal plane of the cell are considered to act as sites of signal transmission. Thus, apical plaques on the free surface side of endothelial cells likely function as sensors for signals directly from the blood flow, and they directly receive dynamic information transmitted to stress fibers [58,59]. We clarified the function and structure of FAK and its associated signal transduction-related proteins that are present in apical plaque. The FAK in apical plaque in endothelial cells seems to play a key role in the determination of elongation along the direction of blood flow. The mechanisms that underlie the recognition of mechanical stimuli by blood flow remain to be elucidated [59].

The stress fibers that are distributed on the upper surface of the cell are connected to the apical plasma membrane, together with highly electron-dense material detected by electron microscopy [60]. Moreover, an analysis of three-dimensional optical sections by confocal laser scanning microscopy together with electron microscopy indicated that the highly electron-dense material consisted of various adhesion-related proteins (actin, myosin, α-actinin, vinculin, talin, paxillin, FAK, integrins, etc.), which were shown to be linked to the apical cell membrane [60]. These results indicated that the focal adhesion-like structure that was present on the basal surface of the cell was also present in the upper surface of the cell with the stress fibers. These apical plaques could be clearly distinguished from other superficial plaque structures [60]. Apical plaque and focal adhesions both include integrin and FAK and, therefore, apical plaque is an essential candidate for sensing mechanical stimuli at the outer surface of the cell. Furthermore, stress fibers running on the upper surface of the guinea pig abdominal aorta vascular endothelial cells as well as the cells in the culture are linked to the cell membrane via focal adhesion-related protein [58]. These observations suggested that the structures connecting stress fibers and the cell membrane, which mainly consist of adhesive proteins, including FAK, may function as mechanical cell stimulation receptors [62]. This study revealed the binding mode with the cell membrane of stress fibers distributed in the upper surface in fibroblastic cultured cells or endothelial cells in situ, and suggested that they might act as flow receptors in vascular endothelial cells.

The mechanical signals from the extracellular matrix and cellular geometry regulate the nuclear translocation of transcriptional regulators, such as Yes-associated protein (YAP), which was recently identified as a mechano-transducer. Cells with FAK knockdown also displayed a low total YAP level, which suggested that cell–ECM adhesion proteins may also regulate YAP expression and/or stability [63]. FAK inhibition led to increased focal adhesion area and actomyosin contractility. The inhibition of FAK leads to focal adhesion maturation and/or mechanical force on focal adhesions, which then promote YAP activation [63,64]. In durotaxis, the cells move on a substrate guided by rigidity gradients and this mechano-signaling is transduced by FAK [65]. Durotaxis requires individual focal adhesions to sample local substrate rigidity and FAK signaling is critical in this process [66]. 

## 4. Morphology Change and Elongation of FAK Knockout Cells

FAK is known to alter cell shape through various ways. RhoA/Rho kinase (ROCK), cytoskeletal dynamics, and FAK are required for mechanical stretch-induced tenogenic differentiation of human mesenchymal stem cells [67]. FAK plays a key role in focal adhesion turnover by transiently inactivating ROCK [36,37].

The stress fibers that comprise the actomyosin system observed in various cultured cells are cytoskeletal structures that are commonly found at sites involved in the generation of contractile force both in vivo and in vitro, and they are exposed to sustained mechanical stimulation applied by blood flow in endothelial cells in the aortae or veins [68,69]. In addition, focal adhesions are structures involved in connecting cells to the substrates on the basal plane, and focal adhesions and the ends of the stress fibers are connected on the plasma membrane via focal adhesion-associated proteins. Two types of stress fibers were identified: those located at the peripheral portion of the cell, which are called peripheral stress fibers, and those located at the central portion of the cell, called central stress fibers [70,71]]. The activation of ROCK controls the formation of stress fibers and focal adhesions in the central part of cultured fibroblasts [69,71]. Local accumulation and the activation of FAK are thought to be important for the formation and destruction of focal adhesions, and the activation of FAK regulates the organization of newly formed stress fibers and focal adhesions by ROCK [35]. The functions of FAK at the time of stress fiber and focal adhesion formation were examined, and the results indicated that the activation of ROCK induced the formation of stress fibers and focal adhesions in FAK knockout (FAK-null or FAK^−/−^) cells [35]. The FAK knockout cells (FAK^−/−^) showed an oval morphology. The numbers of stress fibers and focal adhesions in the center of the cells were decreased, and large focal adhesions were observed to form at the cell periphery. The activation of ROCK in FAK^−/−^ cells transiently increased the number of actin filaments in the center of the cell, but did not show typical stress fiber formation, as seen in normal fibroblasts. In addition, the bundles of actin fibers gradually disappeared. Furthermore, the introduction of the full-length FAK gene into FAK^−/−^ cells resulted in cell expansion. In addition, the numbers of stress fibers and focal adhesions that were localized in the center of the cells increased, and the cells showed typical fibroblastic morphology [35]. Phosphorylated FAK (pY397) and phosphorylated c-Src (pY418) were shown to be localized in focal adhesions. On the other hand, no changes in cell morphology or in the formation of stress fibers and focal adhesions in the central part of the cell were seen in FAK^−/−^ cells that were transfected with green fluorescent protein (GFP)-tagged FAK-related nonkinase (FRNK). FAK also plays an important role in the formation of stress fibers and focal adhesions as well as in the regulation of morphology with the activation of ROCK [35].

## 5. Interaction of c-Src and FAK in Cell Proliferation and Elongation

FAK was identified as a signaling molecule involved in the regulation of cellular behavior that results from integrin interactions with the extracellular matrix [72]. Conservation of SH2 and SH3 binding sites in FAK have been determined and shown previously [73]. Recent research established the functional role of FAK as a positive regulator of both cell motility and cell survival [6,21,37,74]. FAK signaling involves its phosphorylation in response to integrin-mediated adhesion to Tyr-397, allowing for interaction with a variety of signaling effectors, including the Src homology 2 (SH2) domain [75,76,77,78]. Src family kinases recruited to the Tyr-397 site phosphorylate two FAK-interacting proteins, Cas and paxillin, which results in the regulation of Rho family GTPases that ultimately contribute to cell motility [77,78,79,80,81]. Cas phosphorylation is involved in downstream FAK signaling events that confer resistance to apoptosis, as well as phosphatidylinositol 3-kinase (PI3K) activation that results from binding to the Tyr-397 site on FAK [74,82].

Cas was first recognized as a tyrosine phosphorylated protein in cells transformed by v-Crk or v-Src [83,84,85], and it contains multiple protein interaction domains, i.e., the N-terminal SH3 domain, a Src-binding domain near the C-terminus, and an interior substrate domain [86]. The Cas SH3 domain interacts with tyrosine phosphatase as well as FAK and the related tyrosine kinase Pyk2, which suggests that it functions as a molecular switch in the regulation of Cas phosphotyrosine level. The Src binding domain contains a proline-rich motif that can interact with the Src SH3 domain and a nearby tyrosine phosphorylation site (pY668 or Py670) that interacts with the Src SH2 domain [79,87,88,89]. 15 tyrosine residues present in Tyr-X-X-Pro motifs, including Tyr-165, Tyr-249, and Tyr-410, characterize the p130Cas central substrate domain, which is the major region of tyrosine phosphorylation. When phosphorylated, most Tyr-X-X-Pro motifs act as docking sites for proteins with SH2 or PTB domains, including the adapter, Crk [15,90].

The Cas substrate domain is a major region of tyrosine phosphorylation and, when phosphorylated, the substrate domain interacts with the Crk SH2 domain [91]. Many Tyr-XX-Pro sites are phosphorylated to mediate Cas interactions with v-Crk and its normal counterpart, the SH2/SH3 adapter, c-Crk [27,81,86,92]. The binding of Crk to Cas may facilitate downstream signaling events via proteins related to the Crk SH3 domain, including SOS, C3G, and DOCK180, which stimulate the guanine nucleotide exchange of Ras, Rap1, and Rac, respectively [93,94,95,96,97]. Cas tyrosine phosphorylation also promotes SH2-mediated interactions with the adapter Nck-1 [77] and SH2-containing inositol 5′-phosphatase 2 [98]. They also act as downstream effectors of Cas signaling. FAK interacts directly with Cas, but it is not primarily responsible for the phosphorylation of its substrate domain, which is evident by the lack of Cas phosphotyrosine in cells deficient in Src activity [27,77,79,99] and in vitro kinase assays showing that FAK has very poor p130Cas kinase activity [100]. Nevertheless, FAK re-expression in FAK^−/−^ cells result in an increase in p130Cas tyrosine phosphorylation during cell adhesion by Src-mediated phosphorylation of the substrate domain of p130Cas. FAK expression, along with Src, was found to be important in achieving high levels of p130Cas tyrosine phosphorylation in COS-7 cells [100]. These observations indicated that FAK functions as a scaffold protein and it recruits Src to phosphorylate p130Cas.

Similar to p130Cas, paxillin is a non-enzymatic docking protein that has been identified as an intracellular tyrosine phosphorylated focal adhesion protein in cells transformed with v-Src [101,102], and it was shown to contain multiple protein interaction domains [103,104]. Paxillin is tyrosine phosphorylated in response to integrin-mediated adhesion [4], and the major sites have been identified as tyrosines 31 and 118 [15,105,106]. Similar to the p130Cas substrate domain, tyrosines 31 and 118 of paxillin are part of the Tyr-X-X-Pro motif and, when phosphorylated, promote cell–cell interaction that is mediated by paxillin and Crk [15,90]. In addition to Crk, these sites can also bind to other SH2-containing signaling proteins, including C-terminal Src kinase (Csk) [107] and p120RasGAP [108]. Csk is a nonreceptor-type tyrosine kinase that phosphorylates a tyrosine residue in the C-terminal region of Src family kinases, which results in their inactivation. In stimulated T cells, paxillin also interacts with the SH2 domain of the Src family kinase, Lck [109]. The overexpression of FAK in chicken embryo cells [15] or re-expression of FAK in FAK-deficient mouse fibroblasts [78] significantly increases the levels of phosphotyrosine in paxillin, whereas mutations affecting FAK-binding result in decreased phosphotyrosine levels [110]. However, similar to its role in promoting p130Cas phosphorylation, FAK might act as a scaffold primarily to recruit Src kinases and phosphorylate paxillin, as indicated by the observation that phosphorylation of Y397 in FAK cannot promote paxillin phosphorylation [15,78]. The catalytic role of the Src family kinases in the phosphorylation of paxillin is consistent with the observation that the paxillin phosphotyrosine levels are elevated in fibroblasts transformed with v-Src [101] and decreased in fibroblasts lacking Src family kinases [19]. However, FAK kinase activity towards paxillin is upregulated through the phosphorylation of FAK-activated loop tyrosine by Src bound to the Tyr-397 site. The expression of the active form of FAK can promote paxillin tyrosine phosphorylation without the recruitment of Src kinase [111].

FAK can associate with a number of different signaling molecules, adaptor proteins, as well as structural proteins. N-terminal domain of FAK associate with integrin cytoplasmic domains or talin binding to the FAK C-terminal domain might be important for the regulation of FAK by integrins. The autophosphorylation site of FAK at Tyr-397 regulates the direct binding of Src-family protein-tyrosine kinases [20,73]. The above observations strongly support the notion that FAK-Src interaction is important for the regulation of the organization of focal adhesions.

In addition to actin polymerization/depolymerization, ligand binding to the integrin receptor causes FAK talin-mediated oligomer formation. Focal adhesions and related proteins connect the ECM and the cytoskeleton inside the cell. The signals are then transmitted to SFK, such as Fyn and c-Src. FAK is then activated and undergoes autophosphorylation. Tyr397 autophosphorylated FAK binds and then activates Src and Fyn, which in turn phosphorylates the FAK-associated proteins paxillin, tensin, and p130Cas [112]. The phosphorylation of FAK at Y397 promotes the formation of FAK–Src complex. Thereafter, direct phosphorylation by FAK of Src Y418 causes the activation of Src [22]. The downstream target of FAK–Src complex is p130Cas, which is one of the adapter proteins and it acts as a docking protein. The binding of an adapter protein to p130Cas facilitates the activation of small GTPases, such as Rap1 and Rac2 [113]. FAK activates Rac, resulting in leading edge elongation, and FAK also regulates the phosphorylation of paxillin and p130Cas localization to focal adhesions [114]. When Tyr925 of FAK is phosphorylated by c-Src, a complex of GRB 2 and SOS is formed and Ras is activated [115]. Activated Src also phosphorylates p180Cas and induces the formation of a protein complex between Crk and DOCK 180 [116]]. This protein complex enhances the membrane affinity of Rac and further activates the above-mentioned kinase pathway [44]. 

These results indicate that c-Src, together with FAK, plays an essential role in the formation of stress fibers and focal adhesions that accompany the activation of ROCK, and in cell proliferation and elongation.

## 6. Recognition of Adhesive and Nonadhesive Micropatterns in Cultured Cells by FAK

FAK-rich focal adhesions seem to represent the boundary between the plasma membrane and suitable ECM proteins, and such focal adhesions also determine the cell orientation and polarity. Although fibroblastic cells select specific substrates for typical cell–substrate adhesion, the mechanisms of initial contact with the specific substrate that regulates their orientation and polarity seems to be determined by the focal adhesions. 

**Figure 2:**
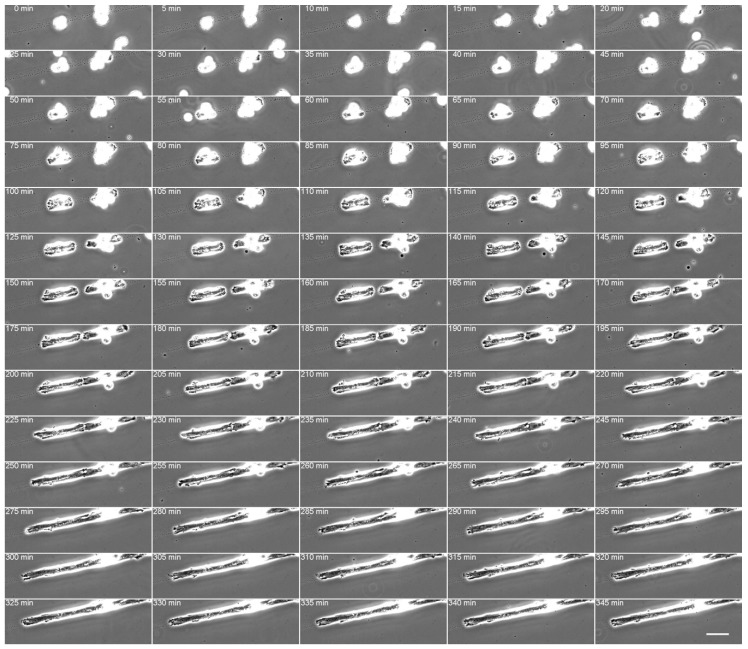
Time-lapse imaging of living fibroblasts adhering to adhesive micropattern (width: 10 µm). The cells were aligned along the longitudinal axis of adhesive micropatterns within 2 h. Adherent cells were not observed on the nonadhesive region of the glass surface. The time intervals are indicated at the top left in minutes. Phase contrast microscopy. See also a supplemental movie for live imaging and Materials and Methods. Scale: 20 µm.

**Figure 3:**
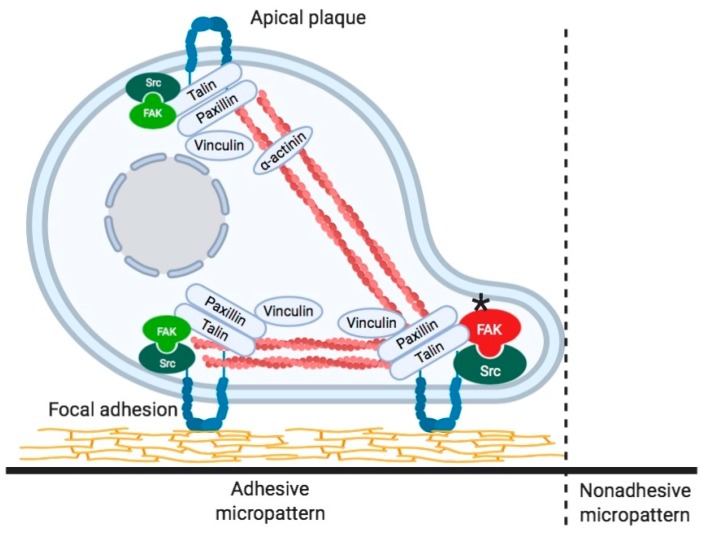
Schematic illustration of a cell adherent on the border of the adhesive and nonadhesive micropatterns. Focal adhesions at the bottom of the cells and the apical plaque are shown. Focal adhesion kinase (FAK) is highly accumulated at the border between adhesive and nonadhesive micropatterns, and the FAK seems to be tyrosine phosphorylated. The asterisk indicates tyrosine phosphorylated active FAK.

Focal adhesions are thought to function in recognition of the state of the cell membrane and the ECM and in determining cell polarity. However, little is known regarding the mechanisms underlying the ECM and intracellular recognition of the ECM by intracellular components and the formation of polarity in the early stages when fibroblasts adhere to the substrate. Micropatterned culture glass (Cytograph; widths of 10 μm) that can control cell adhesion with a very narrow width was used, and the changes in focal adhesion at the initial stage of adhesion and subsequent cell axis formation were examined [117] (detailed materials and methods, see supplemental documentations). When fibroblasts were cultured on micropatterns, small focal adhesion-like structures first formed at the adhesion surface, and the cells eventually began to elongate as spindles along the boundary between the adhesive micropattern and the nonadhesive region. Many small focal adhesion-like structures were formed at the distal end of the cell undergoing extension, and well-developed focal adhesions were formed at the boundary of the micropattern. Furthermore, the accumulation of FAK along the longitudinal direction of the cell was observed at the boundary, which suggested that FAK is essential in determining the cell axis. Our observations imply the presence of phosphorylated c-Src at the boundary. Cells spread sparsely on normal glass surfaces, while the cells only adhered to the micropatterned adhesive surface on micropatterned culture glass. When fibroblasts were spread on micropatterned culture glass about 10 μm in width, they extended along the adhesive surface (Figure 2 and Figure 3, supplemental movie). The adherent cells were longitudinally aligned along the border of the adhesive and nonadhesive regions. When fibroblasts adhered to an ordinary glass surface, the cells first spread into a round shape and extend in a fan shape or an inverted triangle toward the direction of travel after about 1–2 h. These observations indicated that cells could be artificially extended in the long axis direction while using micropatterned culture glass. The nucleus was located at the center of the extended cell, which indicated that the cell was stretched in the longitudinal direction with equal force at both poles.

Adherent cells were longitudinally aligned along the border of the adhesive and nonadhesive regions of the micropattern, and with the signal from anti-FAK antibody highly localized to the border. FAK is a tyrosine kinase and it is one of the proteins constituting focal adhesions. FAK is thought to be a kinase that regulates the integrin and Src families, transmembrane adhesion proteins, and regulates focal adhesion formation and proliferation [26,35,117]. Extracellular matrix information that is received from integrins is transmitted to FAK and induces its phosphorylation. The phosphorylation of FAK is thought to play an important role in regulating cell adhesion, cell migration capability, and wound healing. FAK acts as a regulator of its own formation during the organization of focal adhesions, and accumulation of FAK on micropatterned adhesive surfaces and non-adhesive surfaces is important for fibroblast recognition of adherent and nonadherent sites. 

These observations suggested that FAK itself undergoes tyrosine phosphorylation at the boundary between the adherent and nonadherent surfaces of the cells, which shows that tyrosine phosphorylation of FAK affects recognition of the boundary between the adhesive micropattern and the nonadhesive region. The activation and deactivation of FAK also play key roles in the regulation of elongated cell shape and highly regulated stress fiber organization. FAK has been suggested to play a role in the confinement of cells by adhesive micropatterns by affecting cell polarity. Therefore, the localization of FAK seems to play a key role in the recognition of the border of the cell with the adhesive micropattern, thus regulating cell polarity and the cell axis.

## 7. Conclusions

FAK is a nonreceptor type tyrosine kinase that consists of about 1030 amino acids that regulates integrins and growth factor signals and plays important roles in cell proliferation, differentiation, and apoptosis [118]. FAK undergoes autophosphorylation at tyrosine 397 upon stimulation by integrins and growth factor signals [119], and other phosphorylation sites subsequently undergo phosphorylation to transmit signals downstream to AKT and MAPK [120]. FAK is involved in a number of cell behaviors, including cell proliferation, survival, and invasion, and is therefore thought to play a critical role in the characteristics of malignant tumors. Indeed, FAK has been reported to be involved in various neoplastic diseases, including breast cancer, thyroid cancer, ovarian cancer, head and neck cancer, liver cancer, pancreatic cancer, and lung cancer. The expression status of FAK in cancer is closely related to tumor development and clinical prognosis, but the downstream signals that underlie the roles of FAK in these diseases remain to be elucidated.

## Supplementary Materials

The following are available online at https://www.mdpi.com/2073-4409/9/1/192/s1, Video S1: Video-adhesion-01, Document S1: Materials and Methods.
