# Supplementary material for: FAK-Dependent Cell Motility and Cell Elongation
Source: Cells. 2020 Jan 12;9(1):192. doi: 10.3390/cells9010192 (PMC7017285; doi:10.3390/cells9010192)
Supplement: Supplementary file 1 [file cells-09-00192-s001.zip › Supplementary materials/Materials and Methods.pdf]

1. Materials and Methods

1.1. Cell culture

Fibroblasts (NIH 3T3) were cultured using a 1:1 mixture of Dulbecco's modified Eagle's medium and nutrient mixture (F-12; Gibco, Grand Island, NY), pH 7.4, containing 50 units/mL of penicillin, 50 μg/mL of streptomycin, and 10% fetal bovine serum (Gibco). The cells were maintained at 37°C in a humidified, 5% CO_2 atmosphere.

1.2. Cell adhesive micropattern substrate

Micropatterned cell culture dishes were purchased from Matsunami (CytoGraph; DNP, Tokyo, Japan). The glass surface micropatterns were printed alternately with hydrophobic or hydrophilic printing patterns with intervals of 10, 15, 30, or 60 μm. On a hydrophobic region, the cells do not adhere to the glass surface, thus allowing the control of cell proliferation and cell elongation in a limited space. Living fibroblasts harvested on adhesive micropatterns were observed under phase contrast microscopy (IX-70; Olympus, Tokyo, Japan).
